# Paradoxical Shock and Management of a Post-percutaneous Mechanical Thrombectomy Device in a Morbidly Obese Patient With a Submassive Pulmonary Embolism

**DOI:** 10.7759/cureus.45596

**Published:** 2023-09-20

**Authors:** Soroush Yazdani, Francin Alexis, Keahan Mokhtari, Shahrokh E Rafii

**Affiliations:** 1 Internal Medicine, Brookdale University Hospital Medical Center, New York City, USA; 2 Internal Medicine, St. George's University School of Medicine, St. George's, GRD; 3 Internal Medicine, NewYork-Presbyterian Brooklyn Methodist Hospital, New York City, USA; 4 Medical Student, St. George's University School of Medicine, New York City, USA; 5 Cardiology, Brookdale University Hospital Medical Center, New York City, USA

**Keywords:** submassive pe, impella device, impella rp, percutaneous mechanical thrombectomy, morbidly obese, pulmonary embolism (pe), pulmonary thrombectomy, submassive pulmonary embolism

## Abstract

A submassive pulmonary embolism (PE) is a type of PE where the pulmonary artery is partially obstructed. It is categorized as an intermediate risk when compared to massive PE, which presents as a complete obstruction of the pulmonary artery, therefore placing it in the high-risk category. In either case, if not promptly assessed and treated, it can prove to be fatal. We report the case of a morbidly obese middle-aged female who presented with a submassive PE. Based on the evaluation of the patient's pre-existing conditions, risk factors, clinical severity, imaging, and lab findings, it was concluded that percutaneous mechanical thrombectomy (PMT) was essential to promptly alleviate the clot burden. Following the procedure, it was observed that the patient became hemodynamically unstable, accompanied by hypoxemia and respiratory acidosis. With the assistance of pressors and later the placement of a right ventricular Impella device, the patient was successfully stabilized and, several days later, discharged from the hospital. This report explores the potential factors that may have contributed to the patient's hemodynamic instability and acute right ventricular failure after the PMT procedure. These factors can be attributed to pre-existing changes in the right ventricle (RV) as a result of morbid obesity, as well as possible associations with obstructive sleep apnea or obesity hypoventilation syndrome. Furthermore, it is important to highlight that patients exhibiting submassive PE can be considered suitable candidates for PMT, with careful consideration of the patient's medical history, clinical severity of symptoms, and diagnostic findings. It is worth noting that PMT intervention in this patient demonstrated a favorable outcome.

## Introduction

Pulmonary embolism (PE) is one of the most life-threatening health conditions [1]. After cerebrovascular accidents and myocardial infarction, it is the third most common cause of cardiovascular death. Reports illustrate that PE is responsible for 5%-10% of in-hospital deaths [2]. In the United States, despite decreased deaths from PE in recent years, there are still 100,000 deaths per year [3-4]. Underlying genetic conditions, acquired conditions, and acquired hypercoagulable states are the common causes of PE [5]. Acute PE is categorized into three types: low-risk, submassive, and massive. This categorization is based on hemodynamic stability and parameters for the right ventricle (RV) function. In low-risk PE, patients are normotensive without RV dysfunction. In submassive PE, patients are hemodynamically stable with RV dysfunction. In massive PE, patients are hemodynamically unstable with RV failure [6]. Massive, submassive, and low-risk PE have a mortality rate of 25%-65%, 3%-15%, and <1%, respectively [2]. Depending on the type of PE, different managements are recommended, such as anticoagulation, systemic thrombolysis, catheter-directed thrombolysis, and percutaneous mechanical thrombectomy (PMT). It is reported that PMT provides immediate hemodynamic improvements, symptom reduction, and cardiac function recovery for both massive and submassive PE [7]. We are reporting a case where a morbidly obese patient with submassive PE became hemodynamically unstable immediately after a successful PMT. We suspect that morbid obesity was a major contributor to this transient decompensation.

## Case presentation

**Figure 1:**
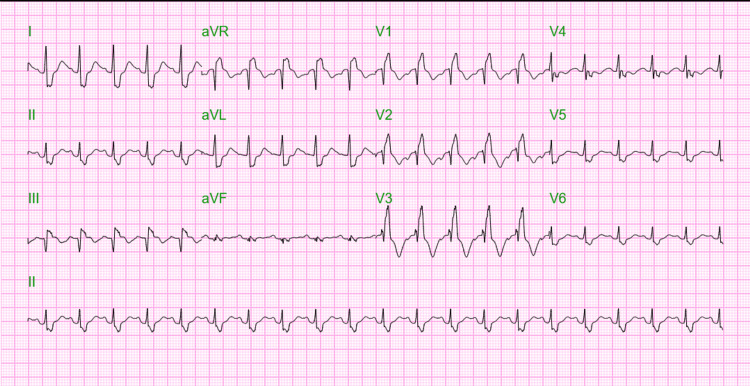
The ECG recorded on the incident day indicates sinus tachycardia, right bundle branch block, and right axis deviation.

A 41-year-old woman with a medical history of morbid obesity (body mass index (BMI): 62.96 kg/m²), hypertension, iron deficiency anemia, and pseudotumor cerebri presented to the emergency room with a sudden onset of dyspnea. The patient exhibited tachycardia and tachypnea and was placed on a non-rebreather mask. The electrocardiogram (ECG) revealed sinus tachycardia, right bundle branch block, and right axis deviation (Figure 1).

**Figure 2:**
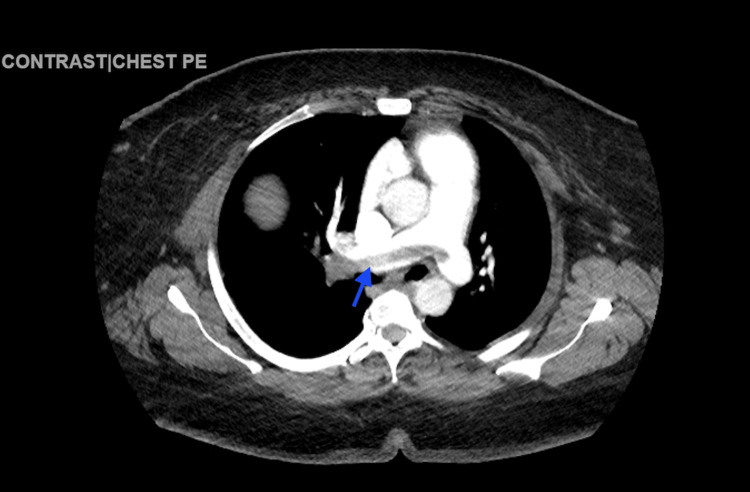
Computerized tomography angiography of the chest with contrast reveals extensive saddle pulmonary embolism (blue arrow).

**Figure 3:**
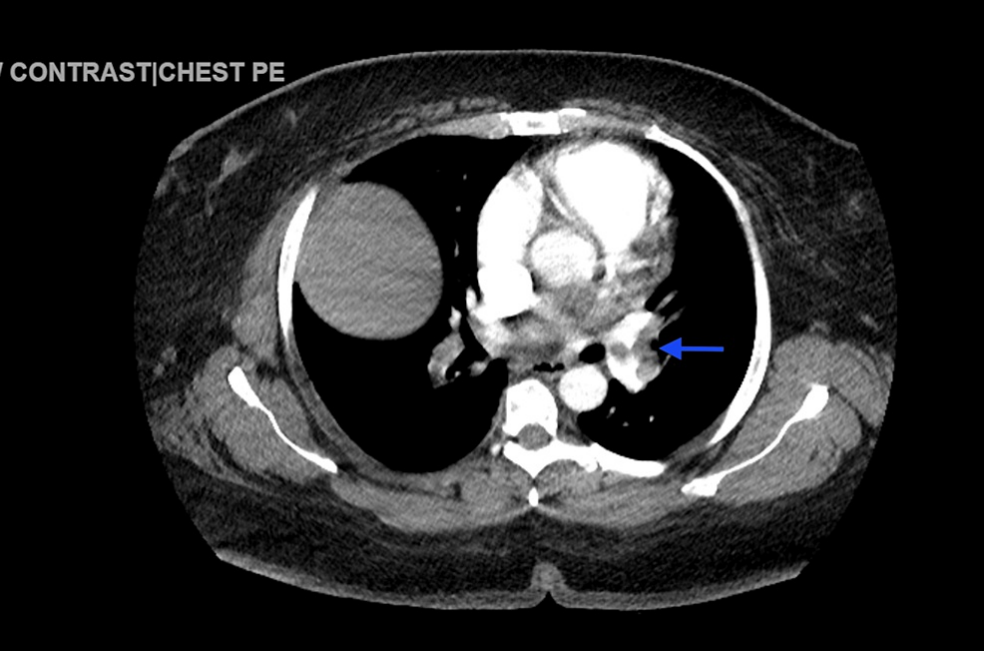
Computerized tomography angiography of the chest with contrast reveals filling defects in the left main pulmonary artery (blue arrow).

**Figure 4:**
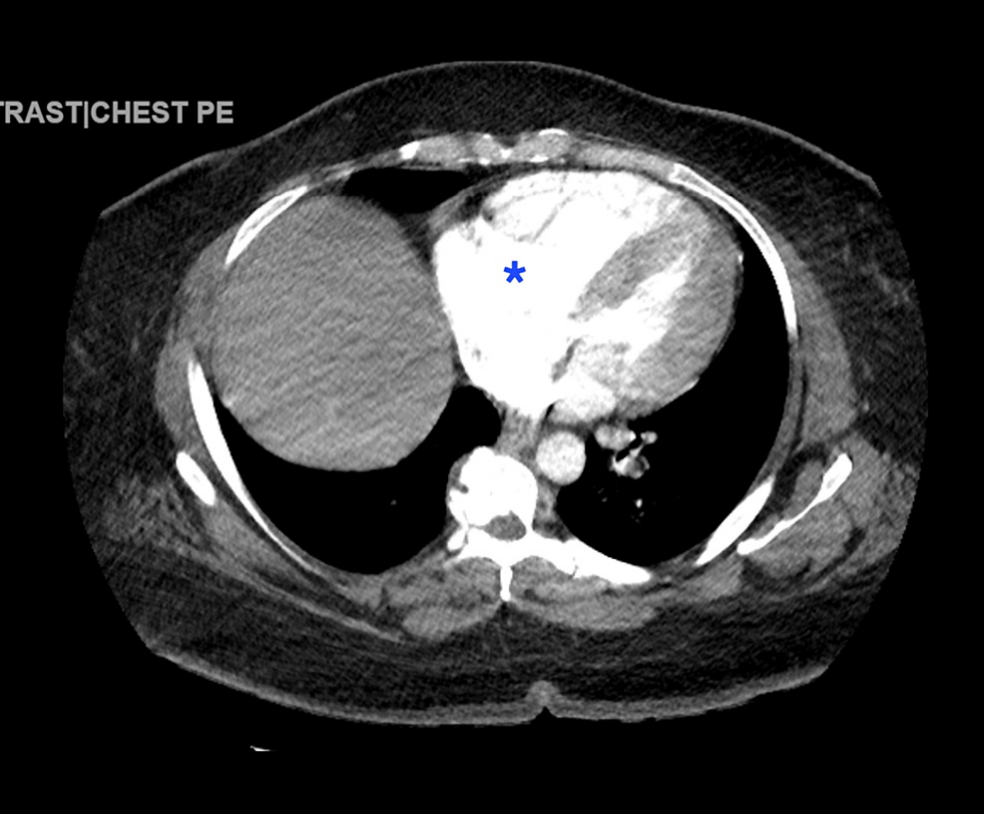
Computerized tomography angiography of the chest with contrast reveals strain in the right ventricle (blue asterisk).

D-dimer levels of 1,137 ng/mL (<0.50 ng/mL), pro-B-type natriuretic peptide (pBNP) of 69 pg/mL (<125 pg/mL), and troponin of 69 ng/mL (0-0.04 ng/mL) were also reported. The computed tomography angiography (CTA) revealed an extensive saddle PE, accompanied by a dilated RV and signs of right heart strain. Additionally, filling defects were observed in the left main pulmonary artery (Figures 2-4).

Full-dose anticoagulation enoxaparin (Lovenox) was given, and the cardiology team was consulted. The patient was transferred to cardiac catheterization for a pulmonary angiogram and PMT and was electively intubated. Arterial blood gas analysis revealed mixed metabolic and respiratory acidosis (acid-base balance of the blood (pH): 7.05, partial pressure of carbon dioxide (pCO_2_): 65.8 mmHg, partial pressure of oxygen (pO_2_): 71.0 mmHg, and serum bicarbonate (HCO3): 18.1 mEq/L). Blood pressure before catheterization was 134/104 mmHg, heart rate was 138 beats per minute, and respiration rate was 28 breaths per minute. Bilateral PMT was completed successfully.

Post-procedure, the patient was noted to be desaturating despite the evacuation of the thrombus. The patient developed hemodynamic instability, along with hypoxemia and respiratory acidosis. In response, phenylephrine boluses were promptly administered, and a norepinephrine bitartrate (Levophed) drip was initiated to achieve hemodynamic stabilization. The RV failure was identified through the measurement of the Pulmonary Artery Pulsatility Index (PAPi), which registered at 0.69, falling below the normal range of >0.9. Consequently, a 23-Fr Impella RP sheath was placed in the RV, contributing to the recovery of the PAPi to a normal value of 1.2. Intravenous heparin was given via Impella. Cardiac thoracic surgery was consulted, and she was not a candidate for extracorporeal membrane oxygenation (ECMO) because of her morbid obesity. Pre-PMT pressure readings were right atrium (RA): 26/27/25 mmHg (normal range (N): 1-8 mmHg), RV: 82/14/33 mmHg (N: 15-30/1-8/8-19 mmHg), and pulmonary artery (PA): 81/37/52 mmHg (N: 15-30/4-14/9-16 mmHg). Post-PMT pressure readings were RA: 15/13/13 mmHg, RV: 34/12/18 mmHg, and PA: 32/26/29 mmHg. Post-thrombectomy transthoracic echocardiography (TTE) revealed RV dysfunction and enlargement in size, and RV systolic function was reduced with mild tricuspid regurgitation. Admitted to the critical care unit after the thrombectomy, and the Impella sheath was successfully removed two days after the procedure. The patient was extubated and discharged from the hospital eight and 13 days post-thrombectomy, respectively, with a prescription for warfarin.

**Figure 5:**
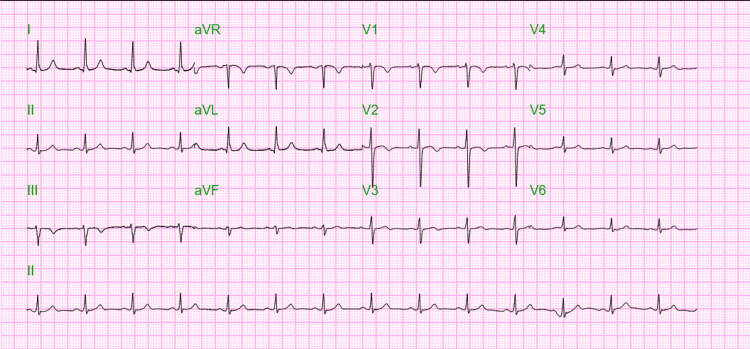
The ECG obtained seven days after the thrombectomy demonstrates a return to normal sinus rhythm.

Figure 5 illustrates the ECG obtained seven days post-thrombectomy, demonstrating a return to normal sinus rhythm.

## Discussion

In 1993, the number of PE admissions in the United States was roughly 60,000. In 2012, more than 200,000 patients were diagnosed with PE [8]. Although the true incidence of PE is unknown, it has been estimated that in the United States, a diagnosis of venous thromboembolism (VTE) is 117 per 100,000, but the true incidence is likely to be higher since these diseases are frequently undiagnosed or are only diagnosed during an autopsy [9]. Risk factors for VTE can be divided into inherited and acquired factors. Acquired risk factors that contribute to the formation of venous thromboembolism tend to be due to trauma, surgery, malignancy, immobility, age, pregnancy, estrogen therapy, and obesity.

Studies have shown that patients with severe obesity (BMI ≥ 35) have a six-fold higher risk of VTE compared with those of normal weight [10]. The mechanism of increased development of VTE is not completely understood, but obesity may predispose an individual to venous stasis. Obesity also has effects on the coagulation system, which include impaired fibrinolytic activity and elevated plasma concentrations of clotting factors such as D-dimer, fibrinogen, factor VIII, and factor IX [11]. Furthermore, obese patients tend to have higher levels of C-reactive protein due to the release of pro-inflammatory mediators by adipose tissue, thus causing a hypercoagulable state and an increased risk of thrombus formation.

Pulmonary embolism is categorized into three groups based on the effects of RV physiology. Massive PE implies hemodynamic instability from RV failure. Submassive PE patients may clinically be normotensive but have evidence of RV dysfunction via echocardiography or CT imaging. Patients with submassive PE have a mortality rate of 3% to 15% [2]. Finally, low-grade PE patients are those who are normotensive with normal RV function.

The physiologic and clinical consequences of PE vary. Symptoms range from asymptomatic to hemodynamic collapse and death. Hypoxemia is the most common physiologic consequence of acute PE. Patients may also experience tachypnea due to medullary receptors sensing the increased carbon dioxide in the body and, in turn, increasing minute ventilation [12].

Initial therapies regarding acute PE center around respiratory support, hemodynamic support (in the form of fluids and vasopressors), and empiric anticoagulation [13]. The standard definitive treatment for PE is divided into hemodynamically stable and hemodynamically unstable patients. Hemodynamically stable patients will usually be given anticoagulation. If there are contraindications for using anticoagulants, an inferior vena cava filter may be warranted. If the clinical severity persists despite the use of anticoagulants, a surgical or catheter thrombectomy or embolectomy may be necessary [14]. For hemodynamically unstable patients, more aggressive treatment would need to be done in the form of thrombolytic therapy [13]. If this treatment is deemed unsuccessful, standard protocol calls for a thrombectomy or embolectomy to be performed.

We focus our discussion on a patient who had a PMT with submassive PE. This is a unique case because the patient in question developed shock after the procedure. Although submassive embolisms typically do not warrant a PMT, it was needed in this case due to the echocardiogram findings and the significant symptoms the patient was experiencing.

Regarding this case, it was expected that her hemodynamic status would improve following a successful PMT; however, despite this release of a large clot burden, she continued to decompensate paradoxically, displaying signs of hypotension and requiring a high dose of a vasopressor. It was noted that she had severe RV failure. This was likely due to her morbidly obese status, which could have played a role in the acute nature of her right heart failure. In fact, studies have shown that overweight and obese individuals without a history of cardiovascular disease can have an increased RV mass, larger RV volumes, and a reduced RV ejection fraction known as cardiomyopathy obesity; therefore, in a post-embolic state, individuals who are overweight or obese may be more susceptible to developing signs of RV failure [14].

In a study done by Sokmen, A. et al., they had three groups of people separated by their respective BMIs. Group 1 was patients who had a BMI<25 kg/m^2^, Group 2 had a BMI of 30-34.99 kg/m^2^, and Group 3 had a BMI > 35 kg/m^2^. The three respective groups underwent TTE, and multiple factors were measured beyond a typical TTE. The results were calculated, and the study determined an increase in RV myocardial performance index was more pronounced in patients with a BMI ≥ 35 kg/m^2^. This finding was most likely due to subclinical impairment of RV systolic function in addition to RV diastolic dysfunction in severe obesity. The early detection of impaired RV function may be important in obesity management [15].

These changes in RV structure could be the result of multiple factors. Obesity poses the strongest risk factor in many other etiologies that can contribute to increased pulmonary vascular resistance, leading to acute right heart failure. Although not documented in our patient’s history, with a BMI of 62.96 kg/m^2^, it is possible that she may have an underlying condition such as obstructive sleep apnea (OSA) or obesity hypoventilation syndrome (OHS). Obstructive sleep apnea is evidenced to have a 40% prevalence in obese individuals, and the prevalence of OHS is linearly correlated to BMI. Pulmonary hypertension occurs in approximately 50% of OHS patients, as compared to approximately 20% of OSA patients [16]. These two entities have similar pathophysiology; they are both a result of pulmonary artery hypoxic vasoconstriction, which subsequently causes arteriolar remodeling, thereby increasing pulmonary vascular resistance, which can significantly contribute to RV strain and acute right heart failure [16].

Although this patient had a submassive PE, she was a suitable candidate for PMT for a variety of reasons. After assessing the patient’s premorbid conditions, risk factors, clinical severity, and CTA findings, it was determined that a PMT was necessary to promptly remove the clot burden. Furthermore, the patient’s inherent risk factors and hemodynamic instability led to the decision to implant the Impella sheath in the RV. The Impella sheath is a catheter-based miniaturized ventricular assist device. Using retrograde femoral artery access it bypasses the RV and ensures adequate blood flow to help maintain systemic circulation [17]. In fact, multiple recent papers have shown good outcomes and suggest the use of the Impella RP sheath in the setting of RV dysfunction to improve hemodynamic performance, allowing time for RV recovery from various insults [18]. Once the RV recovers, the Impella sheath can be removed.

## Conclusions

This case report highlights the necessity for a comprehensive approach to managing submassive PE in obese patients. Customizing treatments based on individual risk factors, clinical severity, and diagnostic findings is crucial. Choosing surgical intervention over delays or alternative treatments was essential to prevent a worse prognosis. Future research is necessary to enhance protocols for submassive PE in this patient group, ultimately improving care and outcomes.
